# The Role of Clinician-Developed Applications in Promoting Adherence to Evidence-Based Guidelines: Pilot Study

**DOI:** 10.2196/55958

**Published:** 2024-12-31

**Authors:** Madhu Prita Prakash, Aravinda Thiagalingam

**Affiliations:** 1 Department of Haematology, Westmead Hospital, Corner Darcy Rd and Hawkesbury Rd, Westmead, Sydney, 2145, Australia, 61 413787632; 2 Westmead Applied Research Centre, University of Sydney, Sydney, Australia; 3 Department of Cardiology, Westmead Hospital, Corner Darcy Rd and Hawkesbury Rd WestmeadSidney, 2145, Australia, Australia

**Keywords:** computerized clinical decision support systems, acute coronary syndrome, clinical guidelines, chest pain pathway, decision support, coronary, heart, cardiac, cardiology, chest, pain, web-based, app, applications, computerized, guideline, emergency, usability

## Abstract

**Background:**

Computerized clinical decision support systems (CDSS) are increasingly being used in clinical practice to improve health care delivery. Mobile apps are a type of CDSS that are currently being increasingly used, particularly in lifestyle interventions and disease prevention. However, the use of such apps in acute patient care, diagnosis, and management has not been studied to a great extent. The Pathway for Acute Coronary Syndrome Assessment (PACSA) is a set of guidelines developed to standardize the management of suspected acute coronary syndrome across emergency departments in New South Wales, Australia. These guidelines, which risk stratify patients and provide an appropriate management plan, are currently available as PDF documents or physical paper-based PACSA documents. The routine use of these documents and their acceptability among clinicians is uncertain. Presenting the PACSA guidelines on a mobile app in a sequential format may be a more acceptable alternative to the current paper-based PACSA documents.

**Objective:**

This study aimed to assess the utility and acceptability of a clinician-developed app modeling the PACSA guidelines as an alternative to the existing paper-based PACSA documents in assessing chest pain presentations to the emergency department.

**Methods:**

An app modeling the PACSA guidelines was created using the Research Electronic Data Capture (REDCap) platform by a cardiologist, with a total development time of <3 hours. The app utilizes a sequential design, requiring participants to input patient data in a step-wise fashion to reach the final patient risk stratification. Emergency department doctors were asked to use the app and apply it to two hypothetical patient scenarios. Participants then completed a survey to assess if the PACSA app offered any advantages over the current paper-based PACSA documents

**Results:**

Participants (n=31) ranged from junior doctors to senior physicians. Current clinician adherence to the paper-based PACSA documents was low with 55% (N=17) never using it in their daily practice. Totally, 42% of participants found the PACSA app easier to use compared to the paper-based PACSA documents and 58% reported that the PACSA app was also faster to use. The perceived usefulness of the PACSA app was similar to the perceived usefulness of the paper-based PACSA documents.

**Conclusions:**

The PACSA app offers a more efficient and user-friendly alternative to the current paper-based PACSA documents and may promote clinician adherence to evidence-based guidelines. Additional studies with a larger number of participants are required to assess the transferability of the PACSA app to everyday practice. Furthermore, apps are relatively easy to develop using existing online platforms, with the scope for clinicians to develop such apps for other evidence-based guidelines and across different specialties.

## Introduction

### Computerized Clinical Decision Support Systems

Computerized clinical decision support systems (CDSS) are software programs designed to improve health care delivery [1]. CDSS can be utilized across a range of medical domains such as electronic drug prescription, medical imaging, clinical diagnosis, and clinical management. It has the ability to integrate patient specific information with evidence-based guidelines to suggest the most appropriate next step in management. CDSS have been shown to improve patient care [2,3] and the use of such digital guidelines in Australia is increasing [4].

Mobile apps are an example of CDSS that are increasingly being employed in clinical practice, particularly in lifestyle interventions and disease prevention. Furthermore, while developing certain types of CDSS can be complex and involve challenging algorithms, there has been a rise in clinician-developed health informatics that does not require extensive expertise in information technology. The availability of sophisticated and user-friendly web-based platforms (such as Research Electronic Data Capture; REDCap) has enabled clinicians to effectively develop simple CDSS programs. Several recent studies have employed clinician-developed mobile apps and videos as patient education and disease prevention tools with promising results [5-9]. However, clinician-developed apps in acute patient care, diagnosis, and management have not been studied to a great extent.

### The Pathway for Acute Coronary Syndrome Assessment

Chest pain is among the most common emergency department (ED) presentations in New South Wales [10], and acute coronary syndrome is an important differential diagnosis, with high morbidity and mortality. Several evidence-based guidelines for the assessment of chest pain have been introduced to facilitate timely diagnosis and treatment while standardizing practice across various health care facilities [11].

In New South Wales, The Pathway for Acute Coronary Syndrome Assessment (PACSA) is a set of documents designed to standardize the assessment and management of patients with suspected acute coronary syndrome across the EDs in the state [12]. It includes the PACSA Flowchart and Checklist (for the assessment of suspected acute coronary syndrome), and the PACSA STEMI Reperfusion Flowchart and Checklist (for the management of confirmed ST-elevation myocardial infarction; STEMI). These guidelines are designed to help clinicians risk stratify chest pain presentations and provide the most appropriate next step in management. Currently, the PACSA guidelines are paper-based in a flow chart format, available in the ED or on the internet. Despite its availability across the state, the use of these guidelines in the ED is uncertain.

### Our Project

We hypothesized that the flow chart content of the paper-based PACSA algorithm could be delivered as an app, as a more user friendly and acceptable alternative. Furthermore, our PACSA app was developed by a cardiology physician using the survey function of REDCap (a web application) without needing extensive resources, highlighting that such apps can be developed by clinicians with relative ease and be applied across a range of clinical domains.

## Methods

### Developing the PACSA App

The PACSA app that modeled the current PACSA guidelines was developed by a consultant cardiologist with a total development time of less than 3 hours using the survey function of REDCap, a secure online platform. Several patient parameters can be entered on the app, including age, electrocardiogram (ECG) findings, vital signs, and troponin assays, to stratify patients into the low-, intermediate-, or high-risk group. Based on the stratification, a management guide is offered, such as admission for urgent cardiology review or discharge with follow-up in outpatient rapid access cardiology clinics. The app also featured an ECG interpretation guide that outlined high-risk ECG features that should be actively screened for. This was an optional feature that participants could access via an accessory link on the app.

### Setting and Study Population

The study was conducted in the ED of Westmead Hospital, a large tertiary hospital covering a catchment area with a population of over 1.5 million people [13]. Patients presenting to the department are briefly assessed by nursing staff (vital signs are measured, and ECG findings are evaluated if indicated) and subsequently triaged for review by a doctor. The medical staff in the department comprises a wide range of junior and senior medical officers including board-certified emergency physicians (consultants), emergency physicians in training (registrars), junior medical officers (interns or postgraduates in years 1‐3), and assistants in medicine (medical students undertaking early work placement).

### Study Design

The study was conducted as a 2-step process. First, participants (ED doctors) were presented with two hypothetical clinical scenarios of an individual presenting with chest pain. Each scenario outlined the individual’s risk factors, character of chest pain, vital signs, ECG findings, and troponin assays. Participants were asked to compute this information into the PACSA app (easily accessible on their smartphones or computer devices) to risk stratify the individual as being at low, intermediate, or high risk. Subsequently, participants were asked to answer a series of survey questions to assess if the PACSA app offered any advantages over the current document. The study scenarios and an example of the PACSA app workflow are provided in Multimedia Appendix 1.

### Data Collection and Analysis

Data were collected through surveys that were distributed to participants as emails, phone messages, and QR codes available around the department. The results of the submitted surveys were automatically stored on the REDCap platform. Our survey parameters included baseline demographic data, frequency of use of the PACSA guidelines, and comparisons of the PACSA app and the PACSA document with respect to ease of use, ease of access, and efficiency. The survey included a combination of dichotomous questions, rating scales, and open-ended questions for free text responses.

Data were reviewed by one author and analyzed as the mean values of the PACSA app versus the paper-based PACSA documents. The free text comments will be reviewed to determine if the suggested changes are feasible for the next version of the PACSA app.

### Ethical Considerations

Ethics approval was granted by the Western Sydney Local Health District Human Research Ethics Committee for this quality assurance project (ID: 2205‐04 QA).

Our project did not involve patients or the use of patient medical records. The participants were required to base their answers on hypothetical scenarios, and their responses remained anonymous. The participants were informed about the purpose of the study when recruited, and participation in the survey was considered implied consent. The study did not offer any compensation for participants.

All survey submissions were anonymous, and data were stored on the secure web-based REDCap platform. Access to the results was password protected and limited to the researchers.

### Writing of the Manuscript

No generative AI tools or programs were used during the writing of this manuscript

## Results

### Study Population

A total of 31 doctors in the ED responded to our survey. The level of experience of our participants included board-certified emergency physicians (consultants), emergency physicians in training (registrars), junior medical officers (postgraduates in years 1‐3), and assistants in medicine (medical students undertaking early work placement), as shown in Table 1. Over 50% of the participants indicated that they never used the current PACSA paper-based documents in their everyday practice; these results are provided in Table 2.

**Table 1.** Level of experience of doctors who participated in the evaluation of the PACSA app with North American equivalents as footnotes.

Level of experience	N (%)
Consultant^a^	6 (19)
Registrar^b^	7 (23)
Postgraduate year^c^ 3	2 (6)
Postgraduate year 2	8 (26)
Postgraduate year 1	4 (13)
Assistant in medicine^d^	4 (13)

a Consultant is the equivalent of a board-certified emergency medicine physician.

b Registrar refers to a doctor in training in emergency medicine.

c Postgraduate years 1‐3 refers to junior medical officers with 1 to 3 years of work experience.

d Assistant in medicine refers to medical students undertaking early work placement.

**Table 2.** Frequency of use of existing paper-based Pathway for Acute Coronary Syndrome Assessment (PACSA; chest pain pathway) documents among study participants.

Frequency of use	N (%)
Always	1 (3)
75% of the time	2 (6)
50% of the time	6 (19)
25% of the time	5 (16)
Never	17 (55)

### Outcomes

The primary outcome was to compare the utility and acceptability of the PACSA app compared to the paper-based PACSA documents in the ED. The two main parameters employed in assessing this were the perceived ease of use and the perceived usefulness of the paper-based PACSA documents compared to the PACSA app. In total, 13 participants (42%) found the PACSA app easier to use, and 3 participants (10%) found the PACSA app less user-friendly compared to the document. The PACSA app was reported to be “useful” by 14 participants (45%), “somewhat useful” by 10 participants (32%), and “not useful” by 5 participants (16%). This was similar to the paper-based PACSA documents, with 10 participants (32%) reporting it as “useful,” 17 (55%) reporting it as “somewhat useful,” and 3 (10%) reporting it as “not useful.” These results are reported in Table 3.

**Table 3.** Perceived ease of use and usefulness of the paper-based Pathway for Acute Coronary Syndrome Assessment (PACSA; chest pain pathway) documents and the PACSA app.

Survey items	N (%)
Ease of using the existing paper-based PACSA document	
Easy	16 (52)
Somewhat easy	12 (39)
Somewhat difficult	3 (10)
Difficult	0 (0)
Ease of using the PACSA app	
Easy	20 (65)
Somewhat easy	6 (19)
Somewhat difficult	1 (3)
Difficult	2 (6)
No response	2 (6)
Is the PACSA app easier to use compared to the paper-based PACSA document?	
Yes	13 (42)
The same	13 (42)
No	3 (10)
No response	2 (6)
Usefulness of the paper-based PACSA document	
Useful	10
Somewhat useful	17
Not useful	3
No response	1
Usefulness of the PACSA app	
Useful	14
Somewhat useful	10
Not useful	5
No response	2

Other survey parameters included the participants’ views on the accessibility of the PACSA app and the time taken to use the PACSA app. The majority of participants (n=21, 68%) found the PACSA app easy to access. The majority of participants (n=18, 58%) also felt that the PACSA app was faster to use compared to the paper-based PACSA documents. The addition of the ECG interpretation guide was found to be useful by 16 participants (52%). These results are shown in Table 4.

**Table 4.** Additional survey items of the .

Survey items	N (%)
Ease of accessing the Pathway for Acute Coronary Syndrome Assessment (PACSA) app	
Easy	21 (68)
Somewhat easy	6 (19)
Somewhat difficult	1 (3)
Difficult	2 (6)
No response	1 (3)
Is the PACSA app faster to use compared to the paper-based PACSA document?	
Yes	18 (58)
The same	9 (29)
No	2 (6)
No response	2 (6)
Usefulness of the electrocardiogram interpretation guide	
Useful	16 (52)
Somewhat useful	7 (23)
Not useful	5 (16)
No response	3 (10)

The two hypothetical scenarios were risk-stratified by the study authors (which included a cardiology physician) prior to the commencement of the study. Using the PACSA app, correct risk stratification was achieved by 26 (84%) participants for case 1 and by 27 (87%) participants for case 2. Only 2 participants (6%) had the incorrect risk stratification in each case. These results are summarized in Table 5.

**Table 5.** Accuracy of risk stratification using the Pathway for Acute Coronary Syndrome Assessment (PACSA; chest pain pathway) app computer decision support system.

Case	Correct risk stratification,n (%)	Incorrect risk stratification,n (%)	No response,n (%)
Case 1	26 (84)	2 (6)	3 (10)
Case 2	27 (87)	2 (6)	2 (6)

Our survey included a free response section where participants were asked to provide feedback about the PACSA app. Unfortunately, the response rate in this section was poor, and evaluation of this survey item was not possible.

## Discussion

### Principal Findings and Implementation of the PACSA App

The PACSA documents were developed to standardize the management of chest pain presentations across health institutions in New South Wales. However, the results of our study suggest that the PACSA tool is inconsistently used in the ED. Barriers to the uptake of the PACSA tool may include time constraints, the cumbersome nature of accessing paper-based PACSA documents, or a physician’s preference to rely on clinical experience rather than protocol-led management plans.

Our results suggest that the PACSA app may offer a more acceptable alternative with the potential to increase physician adherence to evidence-based guidelines. Participants in our study found the PACSA app easier and faster to use compared to the paper-based PACSA documents. Almost all the participants correctly risk-stratified the clinical scenarios by using the app.

CDSS aid in the evaluation of chest pain and include guidelines from several societies such as the American College of Cardiology and the American Heart Association, with subscription-based databases such as UpToDate [14] or online risk assessment tools such as MDCalc. However, the disadvantages of these resources include cost (with many requiring paid subscriptions) and the inability to incorporate institution-specific protocols. In comparison, our PACSA app was developed by a physician using an easily available online platform and in a relatively short time frame. Furthermore, our PACSA app was customized to local protocols and management guidelines and is freely available.

Some barriers to the implementation of CDSS include poor computer literacy, lack of regular guideline updates, and physician reluctance to use protocol-driven tools during patient consultation due to workflow disruption [15-17]. Strategies to overcome these barriers include the development of user-friendly CDSS that are regularly updated to reflect changes to guidelines. Another key strategy is the integration of a CDSS into clinical workflow that assists the clinician in decision-making without interrupting their patient encounter [18]. Our PACSA app is user-friendly as demonstrated from the results of the study and can be customized to include local contact information (such as the contact information for echocardiography technicians, outpatient cardiology clinics, or the inpatient cardiology teams). Furthermore, additional features on the app can allow ED physicians to directly refer patients to our hospital-based Rapid Access Cardiology Clinic or inpatient cardiology review based on patient risk stratification. This integration of our PACSA app into clinical workflow facilitates communication between ED doctors and specialty teams with the potential to expedite patient care. Moreover, any changes to guidelines can be easily reflected on the app using the same online platform.

### Limitations

A limitation of our project is that it was a single-center study with a small number of participants. Similar studies with a larger number of recruits across multiple institutions are needed for a more rigorous study design. Our qualitative study used surveys to collect rudimentary data as a first step toward implementing CDSS in a busy ED. Further studies with different data collection protocols (such as focused groups or interviews of staff from the ED and cardiology department) may be useful in exploring the limitations of the PACSA app and identifying areas of improvement. Moving forward, a pilot study involving real-world scenarios or multicenter trials would be useful to evaluate the functionality and accuracy of the app and its role in improving patient care. There is scope for further research evaluating the role of CDSS across other specialties and management pathways to support more routine implementation of CDSS across institutions.

### Conclusions

The results of our study indicate that the PACSA documents are currently inconsistently used in the ED for the management of chest pain presentations. CDSS such as the PACSA app can be generated with relative ease by a clinician and are a more acceptable alternative with the potential to improve physician adherence to evidence-based guidelines.

## Supplementary material

10.2196/55958 Multimedia Appendix 1 Hypothetical scenarios and an example of the Pathway for Acute Coronary Syndrome Assessment (PACSA) app workflow for risk stratification.

## Abbreviations

CDSS: computerized clinical decision support systems
ECG: electrocardiogram
ED: emergency department
PACSA: Pathway for Acute Coronary Syndrome Assessment
REDCap: Research Electronic Data Capture
STEMI: ST-elevation myocardial infarction
