# Supplementary material for: The Role of Clinician-Developed Applications in Promoting Adherence to Evidence-Based Guidelines: Pilot Study
Source: JMIR Cardio. 2024 Dec 31;8:e55958. doi: 10.2196/55958 (PMC11706440; doi:10.2196/55958)
Supplement: Multimedia Appendix 1 [file cardio-v8-e55958-s001.docx]

# **APPENDIX 1**

## **Survey Items**

**Figure 1A: hypothetical scenarios provided to participants during the survey**


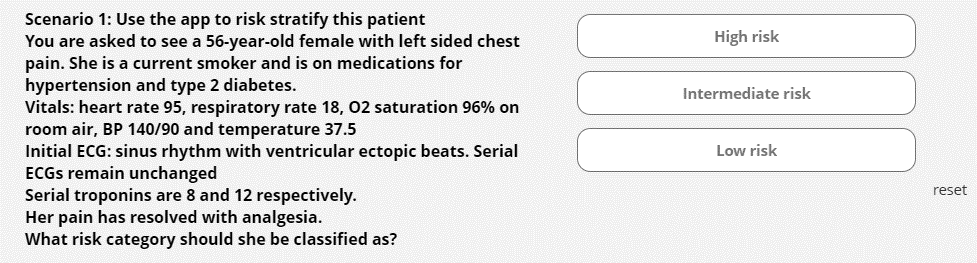


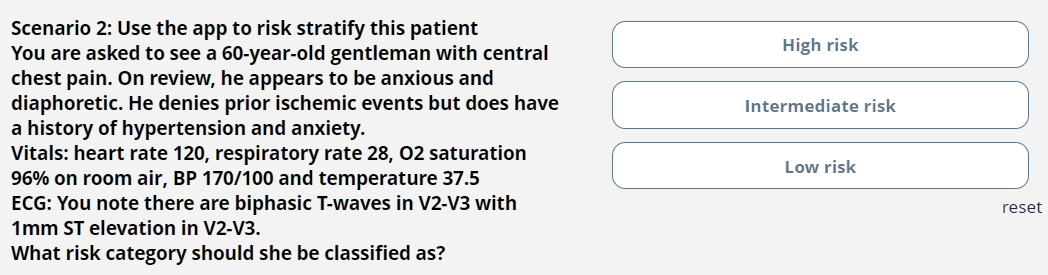


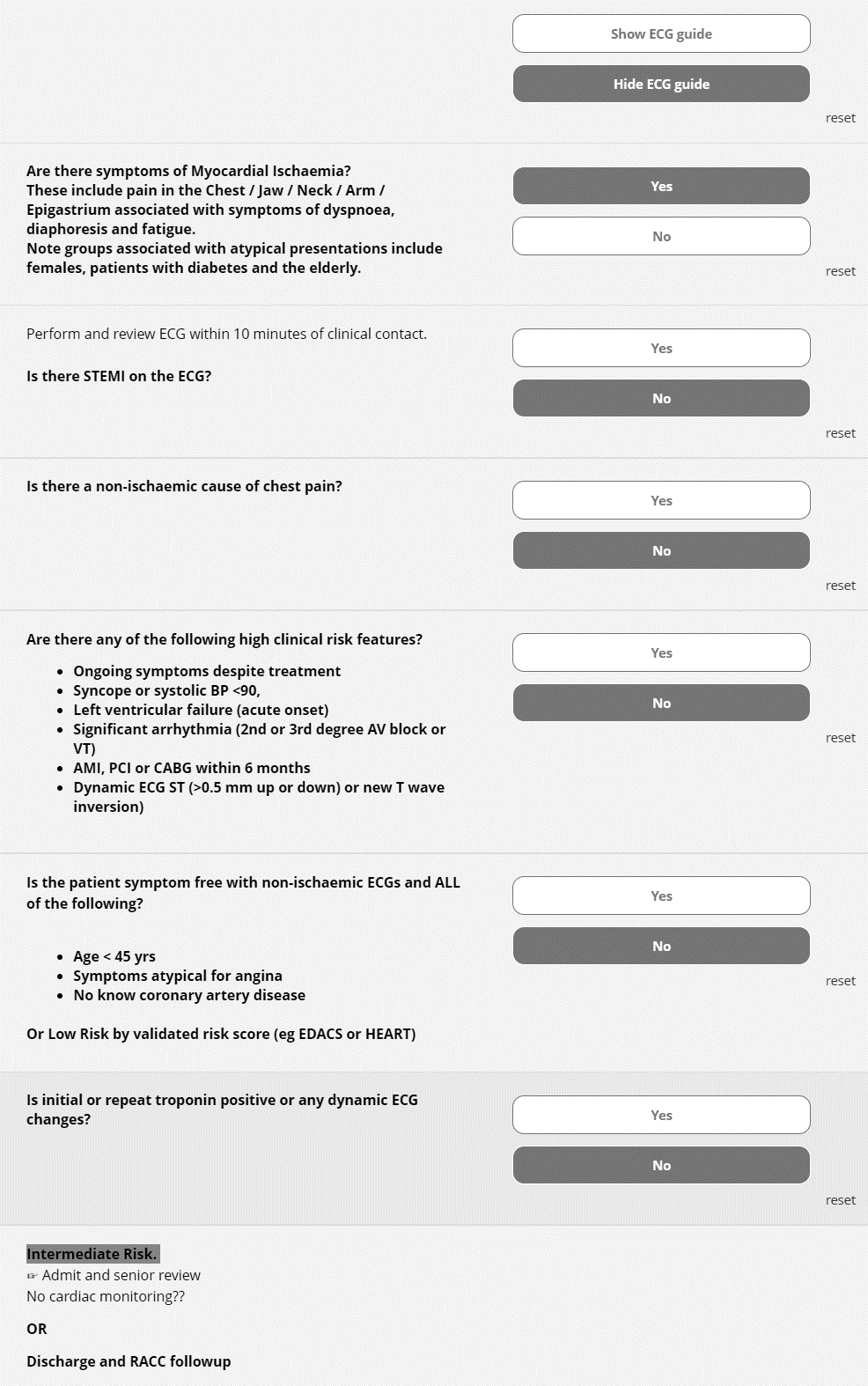
**Figure 1B: An example of the PACA App workflow to risk stratify a patient**
